# The contribution of benchmarking to quality improvement in healthcare. A systematic literature review

**DOI:** 10.1186/s12913-022-07467-8

**Published:** 2022-02-02

**Authors:** Claire Willmington, Paolo Belardi, Anna Maria Murante, Milena Vainieri

**Affiliations:** Institute of Management and Department EMbeDS, Sant’Anna School of Advanced Studies, Pisa, Piazza Martiri della Libertà, 33, Pisa, Italy

**Keywords:** Benchmarking, Quality improvement, Healthcare quality, Process indicator, Outcome indicators, Performance indicators

## Abstract

**Background:**

Benchmarking has been recognised as a valuable method to help identify strengths and weaknesses at all levels of the healthcare system. Despite a growing interest in the practice and study of benchmarking, its contribution to quality of care have not been well elucidated. As such, we conducted a systematic literature review with the aim of synthesizing the evidence regarding the relationship between benchmarking and quality improvement. We also sought to provide evidence on the associated strategies that can be used to further stimulate quality improvement.

**Methods:**

We searched three databases (PubMed, Web of Science and Scopus) for articles studying the impact of benchmarking on quality of care (processes and outcomes). Following assessment of the articles for inclusion, we conducted data analysis, quality assessment and critical synthesis according to the PRISMA guidelines for systematic literature review.

**Results:**

A total of 17 articles were identified. All studies reported a positive association between the use of benchmarking and quality improvement in terms of processes (*N* = 10), outcomes (*N* = 13) or both (*N* = 7). In the majority of studies (*N* = 12), at least one intervention, complementary to benchmarking, was undertaken to stimulate quality improvement. The interventions ranged from meetings between participants to quality improvement plans and financial incentives. A combination of multiple interventions was present in over half of the studies (*N* = 10).

**Conclusions:**

The results generated from this review suggest that the practice of benchmarking in healthcare is a growing field, and more research is needed to better understand its effects on quality improvement. Furthermore, our findings indicate that benchmarking may stimulate quality improvement, and that interventions, complementary to benchmarking, seem to reinforce this improvement. Although this study points towards the benefit of combining performance measurement with interventions in terms of quality, future research should further analyse the impact of these interventions individually.

**Supplementary Information:**

The online version contains supplementary material available at 10.1186/s12913-022-07467-8.

## Background

Introduced in the late 70s as an effort to reduce production costs in the manufacturing sector, benchmarking has since then been used as a method for continuous quality improvement in many different sectors and fields [1]. Although international literature has provided several definitions and taxonomies of benchmarking [2–6], all of them share a common theme, defined as a “continuous process of measuring products, services and practices against the toughest competitors or those companies recognized as industry leaders” [2].

Starting from the 1990s, benchmarking has been applied to the healthcare sector with the aim of measuring and comparing clinical outcomes across organizations as well as enabling them to learn from one another and apply best practices [1, 7]. Benchmarking has become a structured method in the United States and the United Kingdom with the end goal of comparing hospital outcomes for cost-containment purposes [8], although comparison of outcome indicators dates back to the seventeenth century. The increased use of benchmarking was influenced by different factors, including the need to identify and better understand differences in healthcare practices and outcomes between and within different geographical areas [9]. If properly used, benchmarking may also provide a mechanism to detect unwarranted variation and promote the reduction of such [10, 11].

Nowadays, benchmarking represents one of the strategies used for quality improvement, that is, «the changes that will lead to better patient outcomes (health), better system performance (care) and better professional development» [12]. When benchmarking is used to this end, it includes a series of steps such as: identification of best performers through data analysis as well as in-depth (qualitative) investigation of factors that support the observed performance and quality improvement. Performance indicators allow for the conversion of quality to quantifiable metrics that can provide simplified information about a larger area of interest and facilitate comparison across organizations [13, 14]. Depending on the context, the indicators reporting benchmarking data can be aimed at different users with varying decision-making capabilities, ranging from patients to clinicians and policy makers [1, 15]. For instance, comparative performance data of certain clinical processes may lead clinicians to engage in different quality improvement activities such as audit & feedback strategies as well as professional development programs, whereas governments and regional authorities may choose to set policies based on the reporting of certain outcomes [15–17]. Thus, it is crucial that performance indicators convey the right type of information to the right stakeholders. Another key element that contributes to the success of benchmarking is the development of reliable and valid performance indicators that are fit for use [13, 17]. This, however, remains a challenge, especially when it comes to cross-national comparisons as countries may differ in coding and methodologies they use to calculate indicators [14, 18]. Additionally, collaboration between benchmarking participants has also been shown to be a key factor contributing to the successful implementation and use of benchmarking in the healthcare sector [19, 20].

A number of reviews provided evidence that combining benchmarking with public reporting had a limited to moderate effect on quality improvement [21, 22]. However, public comparisons of performance of individuals or organizations could lead to controversy as poorer performers may be discouraged to improve if they feel their reputation has been damaged (e.g. “naming and shaming”) [23–25]. On the other hand, public reporting of performance can also be used to stimulate quality improvement if top performance is emphasized (e.g. “naming and faming”) [26].

What emerges from the existing literature is that there is a continuous and growing interest in the systematic assessment and practice of benchmarking undertaken by healthcare systems and international agencies [13, 27–29]. However, the contribution of benchmarking to quality of care has not been studied extensively.

To investigate this further, we conducted a systematic literature review with the aim of answering to the following research questions:

RQ1: Is there a relationship between the use of benchmarking and quality improvement in healthcare?

RQ2: Can benchmarking combined with additional strategies (e.g. meetings among participants, audit and feedback, use of incentives) further stimulate quality improvement?

## Methods

A systematic literature review was conducted according to the Preferred Reporting Items for Systematic Reviews and Meta-Analysis (PRISMA) guidelines [30].

### Search strategy

To identify articles, we searched the following three databases, PubMed, Web of Science and Scopus. Search terms and keywords were defined according to the current literature on benchmarking. We reported in Additional file 1 the search strategies used for each of the databases along with the number of studies found.

The three databases were searched in January 2021, from their inception date to December 2020. The screening of articles followed a two-step process including: i) screening of titles and abstracts and ii) full text reading. Additionally, the reference lists of relevant articles were scanned to overcome the lack of database search generated articles containing the defined keywords in their title or abstract text.

A quality appraisal of the eligible articles was performed using the quality assessment tool (QATSDD) developed by Sirriyeh et al. for reviewing studies with diverse designs [31] (see Additional file 4). Additionally, we summarized the methodological strengths and weaknesses in the results section.

### Study selection

Our search was restricted to peer-reviewed articles published in the English language. Inclusion and exclusion criteria were defined a priori*.* Articles were considered eligible if they empirically assessed the relationship between benchmarking and clinical outcomes as well as processes across at least two entities over time. We considered healthcare entities at all scales of benchmarking analysis: international, national, and regional level.

While we excluded articles that only focused on the direct impact of public reporting on performance, we considered articles in which benchmarking results were publicly available. Furthermore, we included articles in which the benchmarking participants were the sole decision-makers and users of the benchmarking results. As such, we excluded articles where the decision-making was external to the benchmarking participants, as it is the case for value-based programs in the US or consumers making informed choices. Additionally, we excluded studies that estimated the potential effects of benchmarking on quality through prediction models and those in which the relationship between benchmarking and performance was considered too indirect. We also excluded articles which did not assess performance over time. Finally, we excluded conceptual and theoretical articles as well as review articles, although we did not apply a filter concerning the study design (qualitative versus quantitative) or methodological approach as mixed-methods bring valuable contribution to this research field.

Two reviewers (PB and CW) independently screened titles and abstracts for relevance (see step I in search strategy subsection). Once potentially eligible articles were identified, all four authors independently screened full-text articles for inclusion. Any disagreement between reviewers was resolved through internal discussion and until consensus was reached. Additionally, it is worth noting that the heterogeneity of the studies in terms of methodology, clinical areas and study design was taken into consideration during the undertaking of this systematic literature review.

### Data extraction and analysis

Using a data-charting tool (see Additional file 2 for the list of the variables included), we extracted the following information from the articles: authors; title; year; reported impact of benchmarking; type of quality improvement activity; country; data related to the benchmarking initiative (scale, participation, development, communication and indicators); study design; research question and findings. The data-charting tool was designed collectively as well as piloted by all four investigators (CW, PB, AMM, MV). We performed additional searches using authors sources or institutional webpages when information concerning the benchmarking initiative was missing or not specified in the article directly.

Following Donabedian’s definition of quality [32], we classified the results by process and outcome domains. Due to the high level of heterogeneity between studies in terms of outcomes and methodological designs, we were unable to perform a meta-analysis. However, we provided a synthesis of the resulting evidence.

## Results

### Literature search

**Fig. 1:**
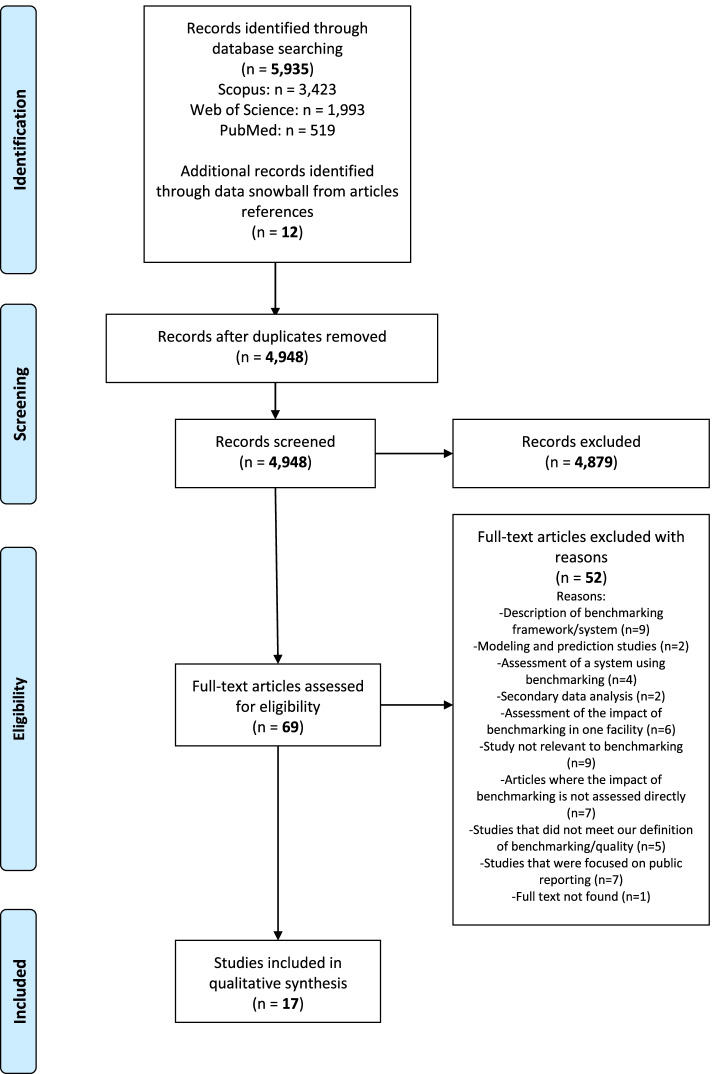
Flowchart of the literature review process

As shown in Fig. 1, the literature search across the three databases identified 5935 articles. An additional 12 articles, identified through scanning of the articles’ references were integrated with the articles identified during the screening of titles and abstracts. Therefore, a total of 5947 articles were identified. The removal of duplicates (*N* = 999) narrowed down the number of articles to 4948. After applying the inclusion and exclusion criteria, a further 4879 articles were excluded from the second round of screening, thus resulting in 69 articles eligible for assessment. Finally, the full-text screening led to the exclusion of 52 articles, reasons being that they either did not meet the inclusion criteria previously defined in the methods section (see subsection “study selection”) or their full texts were unavailable. As such, a total of 17 articles were finally considered for qualitative assessment and synthesis [33–49].

### Study characteristics and benchmarking approaches

Table 1 illustrates the characteristics of the 17 studies. These were published in academic journals between 2004 and 2020 and all benchmarking initiatives were implemented in either North America, Europe or Japan. Thus, all analysed studies took place in high-income countries, as classified by the World Bank [50].

**Table 1:** Description of the studies used in this paper

#	First author; Year	Clinical area	Effect on quality process	Effect on patient outcomes	Type of actions	Benchmarking participants (n)	Units analysed (n)	Benchmarking dimension	Reporting frequency	Number of indicators analysed in the paper	Communication of performance results	Study design
1	Cronenwett et al. 2007 [33]	Surgery - cardiovascular	Preoperative medication use: B-blocker increased from 72 to 91%; preoperative aspirin or clopidogrel from 73 to 83% and preoperative statin from 54 to 72%.	Not evaluated	Biannual meetings attended by different stakeholders (e.g. surgeons, data collection personnel, researchers, and hospital administrators). Participants received instruction in continuous quality improvement techniques and applied these principles to preoperative B-blocker usage.	Hospitals (*n* = 9)	Surgical operations (*n* = 6143)	Quality	Continuous	3	Internal purposes	Observational
2	Campion et al. 2011 [34]	Oncology - Palliative	Higher performance for recurring participants on 9 indicators related to the assessment of pain and dyspnea as well as hospice care.	Not specified	Not specified.	Clinics (*n* = 178)	Clinics (*n* = 178)	Quality	Continuous	15	Internal purposes	Observational
3	Stern et al. 2011 [35]	Cystic fibrosis	Not specified.	Centres improved on indicators related to patient weight and lung capacity over a three-year period.	Best centres asked to define their strategies and share them to feed a learning processes/quality improvement. Open internal discussions. Plan-do-check-act (PDCA) cycles.	Cystic fibrosis centers (*n* = 12)	Cystic fibrosis centers (*n* = 12)	Quality	Continuous	3	Public disclosure	Observational
4	Hermans et al. 2013 [36]	Diabetes	No significant change.	Higher proportion of patients in the benchmarking group reached clinical targets than in the control group over a 12-month follow-up period.	Not specified.	Primary care physicians (*n* = 477)	Primary care physicians (*n* = 477)	Quality	Not continuous	4	Not reported	RCT
5	Merle et al. 2009 [37]	Hip replacement	Indicators related to clinical processes (e.g. time between discharge from orthopedic ward and completion of orthopedic hospitalization record) improved.	Lower percentage of readmissions to acute care in all participating hospitals. Lower percentage of pts. with pressure sores in one hospital. Time to surgery improved in single hospitals.	Review/discussion of comparative performance results by the teams followed by implementation of quality improvement as deemed necessary by each team: improving nutritional status, shorten delays, improving communication btwn professionals.	Hospitals (*n* = 3)	Hospitals (*n* = 3)	Quality, appropriateness and patient safety	Not continuous	15	Internal purposes	Interventional
6	Hall et al. 2009 [38]	Surgery - general	Not specified.	Improvement of both mortality and complication rates across participating hospitals.	Best practices guidelines; case studies of hospitals improving; and rapid data feedback for monitoring progress were provided to participating hospitals.	Hospitals (*n* = 187)	Hospitals (*n* = 187)	Quality, appropriateness and patient safety	Continuous	2	Internal purposes	Observational
7	Tepas III. et al. 2014 [39]	Surgery - general	Not specified.	Reduction of postoperative complications (14.5%): lower incidences of catheter-associated urinary tract infections, surgical site infections, and adverse events after colorectal surgeries in patients over 65.	Monthly participant conference calls.	Hospitals (*n* = 54)	Surgical operations (*n* = 38,896)	Patient safety	Non continuous	4	Internal purposes	Observational
8	Nuti et al. 2016 [40]	Multiple	Performance improvement on composite indicator (encompassing hospital, primary and preventive care) in 11 out of 21 regions.	Not evaluated.	Strategic planning and goal setting of health authorities involved. P4P schemes for heads of health authorities. Communication and discussion of results among different stakeholders including managers, clinicians and patients.	Regional healthcare systems (*n* = 21)	Regional healthcare systems (*n* = 21)	Population health, regional strategy compliance, quality, patient satisfaction, staff satisfaction, efficiency	Continuous	14	Public disclosure	Observational
9	Govaert et al. 2016 [41]	Oncology_colorectal cancer	Not specified.	Severe complication rate and mortality rate declined by 20 and 29% respectively. Length of hospital stay declined by 13%.	Not specified.	Hospitals (*n* = 29)	Patients (*n* = 9913)	Quality, appropriateness and patient safety	Continuous	4	Internal purposes	Observational
10	Piccoliori et al. 2020 [42]	Primary care	Improvement on indicators related to documentation of patient charatersitics, diagnostic tests and prescription of anticoagulants.	Improvement over 1 to 2 years follow-up: Lower percentage of patients with lower blood pressure; Higher number of diabetic patients with HbA1c < 7.0%; Higher percentage of patients with lower LDL-cholesterol.	Self-audit. Technical support provided to participants. Quality circles conducted twice a year to discuss results and strategies for improvement.	General practitioners (*n* = 36)	General practitioners (*n* = 36)	Quality	Not continuous	91	Not reported	Interventional
11	Qvist et al. 2004 [43]	Multiple	Improvement on indicators related to documentation of patient charatersitics, planning of clinical pathway, medication and information provision to patients.	No significant changes.	Conference held btwn two audit rounds. Wards with highest performance gave presentationson local processes of care. Quality improvment projects.	Hospitals (*n* = 47)	Hospitals (*n* = 47)	Quality	Not continuous	10	Internal purposes	Observational
12	Nuti et al. 2013 [44]	Multiple	More than 50% of the indicators significantly improved their yearly performance over the 4-year period.	More than 50% of the indicators significantly improved their yearly performance over the 4-year period, including the percentage of femur fractures operated within 2 days.	Linkage between preformance on indicators and CEO’s reward system. Regular meetings between different stakeholders, including managers and clinicians.	Regional local health authorities (*n* = 12) and teaching hospitals (*n* = 4)	Regional local health authorities (*n* = 12) and teaching hospitals (*n* = 4)	Population health, capacity to pursue regional strategies, clinical performance, patient statisfaction, staff satisfaction, effiency	Continuous	130	Public disclosure	Observational
13	Van Leersum et al. 2013 [45]	Oncology_colorectal cancer	Increase in % of patients discussed in a pre-operative meetings. Improvement inù the implementation of recommended guidelines on preoperative MR-imaging for rectal cance. Improved standard of pathological reporting.	Postoperative morbidity, length of hospital stay and postoperative mortality decreased significantly. The re-intervention rate decreased.	Not specified.	Hospitals (*n* = 92)	Patients (*n* = 24,828)	Quality, appropriateness and patient safety	Continuous	10	Public disclosure	Observational
14	Margeirsdottir et al. 2010 [46]	Diabetes	Use of intensive insulin treatment and pumps increased.	The mean HbA1c of all clinics improved.	Quality meetings and discussions. Provision of clinical guidelines to participating teams at the start of the study.	Clinics (*n* = 25)	Patients (*n* = 5599)	Quality and appropriateness	Continuous	7	Internal purposes	Observational
15	Kodeda et al. 2015 [47]	Oncology_colorectal cancer	Preoperative radiotherapy and chemoradiotherapy became more common. Number of multidisciplinary team conferences increased. Indicators related to specific surgical procedures improved.	Postoperative mortality after 30 and 90 days decreased. 5-year local recurrence rate dropped. Proportion of non-operated patients increased.	Regional and national meetings where points and specific findings are presented and discussed by representatives from all hospitals.	All hospitals in Sweden	Patients (*n* = 29,925)	Quality, appropriateness and patient safety	Continuous	22	Public disclosure	Observational
16	Pinnarelli et al. 2011 [48]	Hip replacement	Not specified.	Proportion of hip operations performed within 48 h increased by 34% for Lazio and 46% for Tuscany.	Workshops for discussion and training organised among regional managers and professionals. Performance on indicators are linked with CEO’s compensation system/DRG reimbursement.	Hospitals in Lazio (*n* = 42) and Tuscany (*n* = 26)	Patients (*n* = 273,320)	Quality	Continuous	1	Public disclosure	Observational
17	Miyata et al. 2012 [49]	Surgery - cardiovascular	Not specified	Improvement of operative mortality and morbidity.	Not specified.	Hospitals (*n* = 99)	Isolated CABG procedures (*n* = 3882)	Quality and patient safety	Continuous	2	Internal purposes	Observational

We found that the studies included diverse clinical areas. Nevertheless, a number of studies can be grouped in similar clinical areas (see column “Clinical area” in Table 1), namely oncological care (*N* = 4), surgical care – general and cardiovascular (*N* = 5) - and chronic illeness care (*N* = 3).

In all but one benchmarking initiative, participation was voluntary as opposed to mandatory. Participants varied from individual clinicians to hospitals. In terms of granularity of the analyses (see column “units analysed” in Table 1), the level of data aggregation ranged from individual procedures and patients to hospitals and regional healthcare systems.

**Fig. 2:**
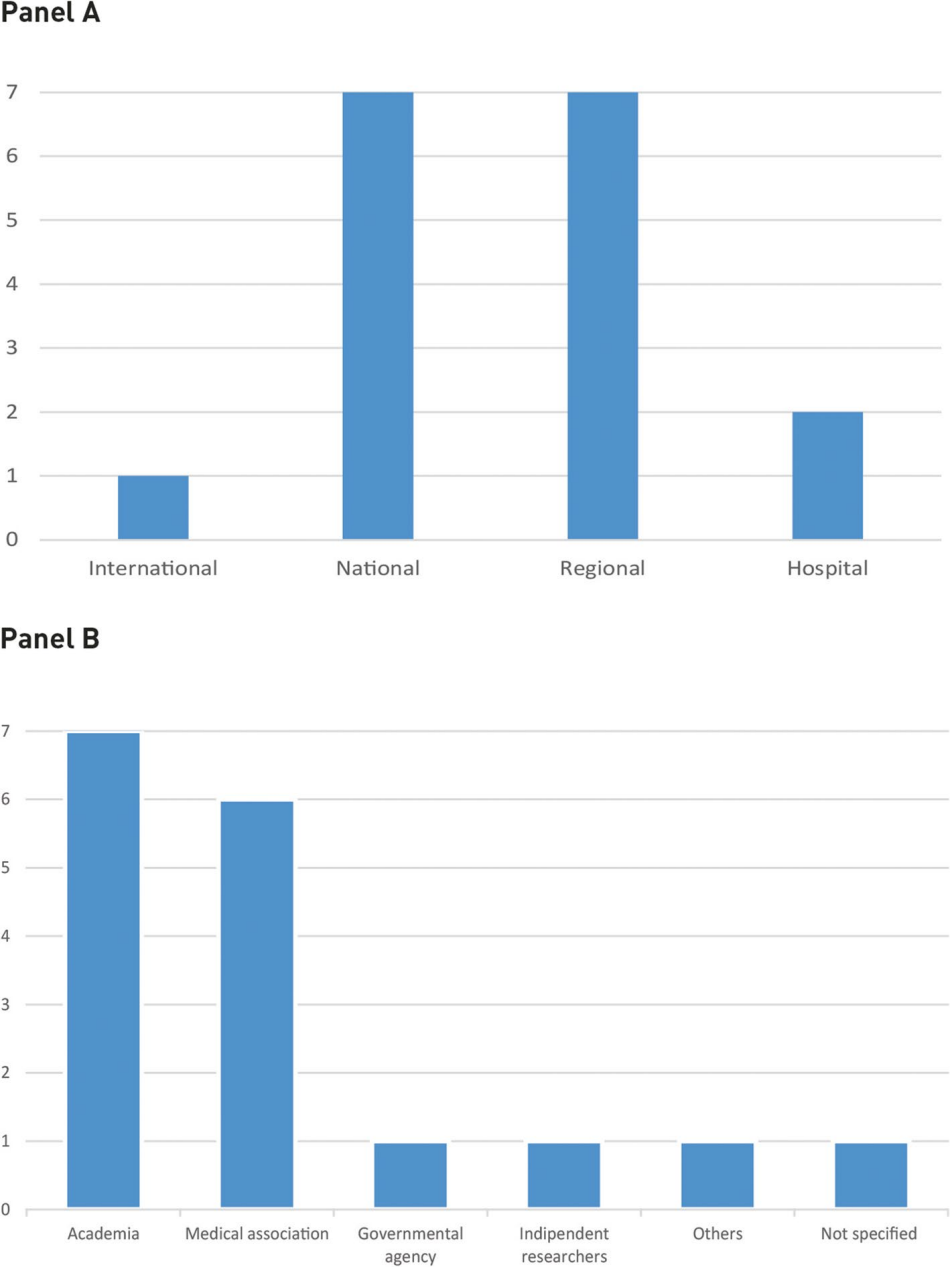
Scale of benchmarking initiatives (Panel **A**) and types of benchmarking developers (Panel **B**)

Figure 2 Panel A illustrates the distribution of the different scales at which benchmarking was carried out. Benchmarking activities were mostly conducted at a national level: either covering an entire territory or selected regions. Only one initiative was implemented at the international level.

As displayed on Fig. 2 Panel B, the benchmarking activities were developed and implemented by a wide variety of actors within the healthcare system. Most of them, however, were carried out by either academia or medical associations (*N* = 13, see studies number 1,2,3,5,6,7,8,9,10,12,13,14,16 in Table 1). Additionally, the majority of benchmarking initiatives (*N* = 11, see column “Reporting frequency in Table 1) monitored performance continuously over time.

With reference to our research objective, we found that all studies included in our analysis reported quality improvement both in terms of care process and outcomes.

Secondly, we found that the use of benchmarking was generally associated with various complementary quality improvement strategies, as illustrated in the following subsections. Finally, all the results reported evidence of a positive contribution of benchmarking, suggesting a bias in the literature.

### Quality improvement in terms of processes and outcomes

Evaluation of performance on process indicators over time was conducted in over half of the studies. Almost all of these studies (*N* = 10) reported significant improvement on these measures. Table 1 shows that measures on medication were most commonly reported (*N* = 4, see studies number 1,10,11,14 in Table 1), followed by measures on documentation of patient’s health (*N* = 3, see studies number 5,10,11 in Table 1), diagnostic test (*N* = 2, see studies number 10,13 in Table 1) and multidisciplinary meetings (*N* = 2, see studies number 8,12 in Table 1). Medication measures included use of B-blockers, anticoagulants and insulin. Six studies did not evaluate care processes (see studies number 3,6,7,9,16,17 in Table 1). Evaluation of performance on process indicators over time was conducted in over half of the studies. Almost all of these studies (*N* = 10, see studies number 1,2,5,8,10,11,12,13,14,15 in Table 1) reported significant improvement on these measures.

14 studies assessed outcome measures over time. Apart from two, all of these studies reported significant improvement on outcome measures, which largely consisted of measures on mortality and post-surgery complications (*N* = 6, see studies number 5,6,7,9,13,15 in Table 1), followed by outcomes for diabetic patients, e.g.systolic blood pressure, cholesterol and HbA1c levels (*N* = 2, see studies number 4,14 in Table 1, hospital length of stay (*N* = 2, see studies number 9,12,13 in Table 1) and time to surgery (*N* = 2, see studies number 5,12 in Table 1). Four of the studies reported adjusted outcome measurements at patient level (age, risk).

Seven studies reported performance improvement on both process and outcome indicators. The study period outlined in all the articles varied from 6 months to 18 years.

Performance changes on process and outcomes indicators reported by each study are described in Table 1.

### Quality improvement related actions

The methods used to improve quality can be classified into two categories: strategies that made direct use of results on performance indicators to actively stimulate performance improvement – audit & feedback, quality improvement plans, Plan-do-check-act (PDCA) cycles, financial incentives - and strategies that indirectly supported quality improvement such as meetings, provision of guidelines as well as technical support.

Table 1 shows that meetings among participants were the most frequently used strategy by benchmarking initiatives to support performance improvement (N = 11, see studies number 1,3,5,6,7,8,11,12,14,15,16 in Table 1), followed by quality improvement plans (*N* = 4, see studies number 1,3,8,11 in Table 1), pay-for performance schemes (*N* = 3, see studies number 8,12,16 in Table 1), provision of guidelines (*N* = 2, see studies number 6,14 in Table 1) and audit & feedback (*N* = 2, see studies number 6,10 in Table 1). A combination of at least two strategies were present in over half of the studies (*N* = 10, see studies number 1,3,5,6,8,10,11,12,14,16 in Table 1). This combination would most commonly include meetings or discussions and direct quality improvement plans (*N* = 5, see studies number 3,5,8,10,11 in Table 1). Additionally, meetings were used as a single strategy in two of the studies. Five studies, on the other hand, did not report any type of quality improvement strategy implemented (see studies number 2,4,9,13,17 in Table 1).

### Methodological approaches for quality improvement measurement

To assess the change in quality linked to benchmarking, most of the studies included in this analysis considered time trends, starting from the beginning of performance reporting (see studies number 1, 3, 6–9, 11–16 in Table 2). Other studies, however, used different approaches, including comparing performance between initial participants and those that joined the benchmarking initiative later (see studies number 2, 17 in Table 2), as well as comparing performance of facilities before and after initiation of benchmarking (see studies number 5, 10 in Table 2). In one case, a control group was used to evaluate the change in performance of facilities that underwent benchmarking (see study number 4 in Table 2). While the articles varied in terms of study periods, ranging from 6 months to 18 years, performance, was on average, monitored over a period of 4 years. The longer the study period was, the more likely information bias was reduced. Seven studies were population-based (see studies number 8, 9, 12, 13, 14, 15, 16 in Table 2), which reduced selection bias in these cases. In certain studies, data was aggregated at the healthcare provider or regional level (see studies number 8, 11,12 in Table 2). Methods for counteracting selection bias and accounting for differences between patients as well as care settings were specified in almost all articles. In certain smaller-scale studies, data analysis was performed and reported for each facility involved, thus also accounting for potential differences between care settings (see studies number 3, 5 in Table 2). In cases where no form of risk-adjustment was performed, the analysis was often focused on process rather than outcome indicators (see studies number 1, 2 in Table 2). Additionally, in two instances, data validation was performed to address information bias (see studies number 6, 13 in Table 2). Aside from one study in which long-term survival was analysed (see study number 15 in Table 2), the majority reported short-term outcomes.

**Table 2:** Summary of methodological strengths and weaknesses

#	First author; Year	Length of follow-up time	Performance evaluation strategy	Patient population	Limitations	Control for biases
1	Cronenwett et al. 2007 [33]	3 years	Time trend	Clearly defined sample of patients undergoing vascular surgery	-Risk adjustment was not performed. -Only processes of care were evaluated.	None specified in the article.
2	Campion et al. 2011 [34]	4 years	Performance compared between initial and later participants	Sample of end-of-life cancer patients defined by age, sex and tumor type	-Risk adjustment was not performed. -Only processes of care were evaluated.	None specified in the article.
3	Stern et al. 2011 [35]	5 years	Time trend	Clearly defined sample of cystic fibrosis patients.	Limited number of care centers involved	-The performance of each center was analyzed separately -Analysis was age-adjusted for certain indicators
4	Hermans et al. 2013 [36]	1 year	RCT	Clearly defined sample of diabetic patients	-Short follow-up time. -Highly heterogeneous group of care settings involved	-Use of control group. -Differences between patients as well as care settings were accounted for in the analysis
5	Merle et al. 2009 [37]	6 months	Before/after comparison	Clearly defined sample of patients undergoing surgical care for hip fracture.	-Short follow-up time -Small number of hospitals involved. -No use of control group	Analysis performed for each hospital involved.
6	Hall et al. 2009 [38]	3 years	Time trend	Sample of patients undergoing general and vascular surgery	-Self selection of centers, thus the results may not be representative of the population. -The analysis is based on sampling.	Different modelling approaches were used to control for differences between patients.
7	Tepas III et al. 2014 [39]	15 months	Time trend	Sample of patients undergoing general and vascular surgery.	-Short follow-up period. -Little information on patient population.	Risk-adjustment was performed.
8	Nuti et al. 2016 [40]	5 years	Time trend	General population	-Highly aggregated data analysis (regional level) -Use of composite indicator that is based on 14 indicators.	-Population-based study -Data was standardized for age and sex
9	Govaert et al. 2016 [41]	3 years	Time trend	-Population-based -Clearly defined sample of patients undergoing surgery for colorectal cancer.	-Only short-term survival was considered.	-Population-based study -Risk-adjustment was performed to account for differences between patients. -External data validation performed
10	Piccoliori et al. 2020 [42]	3 years	Before/after comparison	Sample of patients with chronic conditions.	-Small-scale study -Results were not adjusted for differences between care providers or patients -Little information on patient population	-Information bias was diminished by removing prevalences from the analysis.
11	Qvist et al. 2004 [43]	1 year	Time trend	Few information on patients characteristics as the focus of the analysis is on the providers	-Short follow-up time period -No risk adjustment was performed.	None specified in the article.
12	Nuti et al. 2013 [44]	4 years	Time trend	General population	-Highly aggregated data analysis (regional level)	-Population-based study -Data was standardized for the population’s health needs
13	Van Leersum et al. 2013 [45]	2 years	Time trend	-Population-based -Clearly defined sample of patients undergoing surgery for colorectal cancer.	- Short follow-up time period	-Population-based study -The data was adjusted for differences between patients. -External data validation was performed
14	Margeirsdottir et al. 2010 [46]	5 years	Time trend	-Population-based -Clearly defined sample of pediatric patients with diabetes.	-No information on non-participants	-Population-based study -Adjustment for patient age and duration of disease was performed. -All measurements were standardized.
15	Kodeda et al. 2015 [47]	18 years	Time trend	-Population-based - Clearly defined sample of patients with colorectal cancer.	-Lack of external data validation -Absence of control group	-Population-based study -Longer follow-up time period.
16	Pinnarelli et al. 2011 [48]	3 years	Time trend	-Population-based - Clearly defined sample of patients undergoing surgical care for hip fracture.	-A number of confounders including patient co-morbidities could not be controlled for in the analysis.	-Population-based study -Risk-adjustment of performance was performed.
17	Miyata et al. 2012 [49]	4 years	Performance compared between initial and later participants	Clearly defined sample of patients undergoing coronary artery bypass graft (CABG)	-Limited number of participants involved	-Risk-adjustment of performance was performed

## Discussion

### Summary of main findings

This systematic literature review addresses our research questions by providing evidence concerning a positive association between the use of benchmarking and quality, which is further stimulated when combining benchmarking with specific interventions, such as meetings between participants, quality improvement plans and financial incentives.

The studies we analysed confirm that benchmarking is a useful tool which has yet to be systematically implemented at all levels of the healthcare system [1].

Most of the initiatives were voluntary based and had a bottom-up approach, involving mainly medical associations and academia. More specifically, our findings suggest that benchmarking data was in large part used at the micro level by speciality departments and hospitals, sometimes in the context of small-scale pilot studies that involved a small number of participants [35, 37, 42]. This raises questions regarding the involvement of high-level decision makers when it comes to the use of benchmarking. Importantly, the geographical scope of these studies was limited to Europe and North America.

### Research on the practice of benchmarking

Healthcare systems worldwide are increasingly being called on to identify reliable methods for measuring quality of care [51, 52]. This is partly due to the increasing availability of data generated at all levels of the healthcare system. The practice of benchmarking and performance improvement has been considered, especially in Europe, a growing area of research which has received less attention than the identification of performance indicators that reliably benchmark information in different clinical areas [16].

Following the identification of indicators, the questions ensue as to which users they are intended for and the purpose of their use. The information needs of users may differ depending on their decision-making capacity when it comes to taking action based on benchmarking data. As such, the actionability of this type of evidence-based information remains debatable. Furthermore, certain studies [53, 54] have suggested that benchmarking data was generally underused by decision makers within the healthcare system. On the other hand, when healthcare providers do take into account benchmarking data, reluctance may arise when integrating this information into practice for changing behaviour and procedures [55]. The clinician’s subjective perception can also be a factor when deciding on which areas of performance to consider for improvement [37].

### Benchmarking and quality improvement

All articles considered in this review reported performance improvement following communication of benchmarking data. One could argue, however, that the sustainability of the reported quality improvement could differ from one study to another depending on the length of follow-up time and monitoring of performance. For instance, in five of the articles, performance was monitored over a relatively short period of time, ranging from 6 months to 2 years [36, 37, 39, 42, 43]. Although these studies validate the use of benchmarking as a tool for quality improvement, researchers have argued that, in this case, performance improvement could be attributed to the experimental conditions under which benchmarking is taking place as well as the newness of the initiative itself, rather than a long-lasting impact of performance measurement [49, 56]. On the other hand, articles reporting a longer follow-up time have also shown sustained performance improvement [33, 38, 40, 41, 46]. Interestingly, only one article focused on the capacity of benchmarking to reduce geographical variation [11].

Furthermore, our results suggest that quality improvement was achieved not only by high performing organisations but also by those whose performance was initially suboptimal [38, 39]. It has long been speculated that the combination of continuous performance measurement with interventions, such as discussions of benchmarking results, was associated with long-lasting quality improvement [43, 46, 56]. The majority of articles from our results reported the implementation of these interventions in addition to benchmarking, ranging from meetings to quality improvement plans and audit & feedback. Meetings between benchmarking participants were the most frequently cited intervention by the articles. Although this type of intervention has a more supporting than active role in terms of quality improvement, interactions between benchmarking participants do facilitate direct exchange of experience and transfer of best practices, thus prompting organisations to further engage in activities adapted to their performance needs. Furthermore, our results showed that meetings were often combined with other interventions, such as quality improvement plans and financial incentives. For instance, Italy’s Tuscany region combines discussions of publicly reported benchmarking data between different stakeholders with pay for performance schemes for local decision-makers and clinicians [40, 44, 48]. Although many have recognized the positive effects that benchmarking and quality improvement activities have, some have argued that the extent of their impact on quality remains unclear, and as such, establishing a causal relationship between benchmarking and quality remains difficult [38, 43, 57].

### The relationship between process and outcome indicators

Lastly, several articles included in our review suggest that performance improvement on process indicators is correlated with better outcomes as well, particularly in primary care and certain clinical areas such as diabetes and colorectal cancer [42, 44–47]. This should come to no surprise as it is widely accepted that processes of care contribute in large part to patient outcomes [58, 59]. However, it has been argued that outcomes are reflective of a wide variety of determinants, some related to healthcare and others not. Furthermore, processes of care that are measurable may represent only a fraction of all the processes that contribute to a particular outcome [60]. However, given the ongoing transformation of performance management systems and the rise of innovative measures, including patient reported data, population based indicators and measures on resilience and sustainability [61], one could expect the relationship between processes and outcomes to change.

### Limitations

This literature review included peer-reviewed studies in English, and excluded grey literature as well as foreign language journals. Furthermore, the results show a very limited number of studies on the relationship between benchmarking and quality improvement, despite the growing interest and research on this topic at the international level. Many articles focus on the practical actions to foster benchmarking as a tool to learn from excellence [62], set strategic planning [40, 63], and improve reputation by naming and faming and peer learning [26]. However, these articles provide specific frameworks on the use of benchmarking rather than report results and impacts of its application.

Another limitation relates to the robustness of the methods used as almost all articles are based on observational analysis and are thus susceptible to methodological biases.

## Conclusions

The limited number of studies generated by this systematic literature review suggests that the contribution of benchmarking in healthcare needs to be further explored. Our findings also indicate that benchmarking may foster quality improvement, and that complementary interventions, such as meetings and audit & feedback, can also play a role in further reinforcing quality improvement.

As data becomes more widely available, it is becoming increasingly important for healthcare systems to identify reliable performance indicators that are adapted to the needs of different stakeholders, who ultimately, are the end-users of benchmarking information. As such, further research needs to be conducted as to discern the factors, including contextual elements, that could influence the uptake of benchmarking at all levels of the healthcare system. Although this study points towards the positive impact of combining performance measurement with interventions on quality, future research should analyse the individual impact of these interventions, including non traditional ones such as the promotion of good performance practices.

## Supplementary Information

**Additional file 1.**

**Additional file 2.**

**Additional file 3.**

**Additional file 4.**

## Data Availability

The datasets analysed are available from the corresponding author upon reasonable request.
