# Supplementary material for: The contribution of benchmarking to quality improvement in healthcare. A systematic literature review
Source: BMC Health Serv Res. 2022 Feb 2;22:139. doi: 10.1186/s12913-022-07467-8 (PMC8812166; doi:10.1186/s12913-022-07467-8)
Supplement: Supplementary file 1 — Additional file 1. [file 12913_2022_7467_MOESM1_ESM.docx]

**Additional file 1**

**Title**: search strategies used for each of the databases along with the number of studies found

**Search strategy:**

(benchmarking OR comparison OR "external comparison" OR rating OR ranking OR "comparative evaluation" OR "comparative analysis" OR "comparative assessment" )

AND

("performance tool*" OR "performance project*" OR "performance program*" OR "program performance" OR "performance monitor*"OR “value based program” OR “surveillance program*” OR “surveillance system*” OR “audit of performance” OR “performance audit” OR “control system*” OR “quality control” OR "performance system*" OR "performance evaluation*" OR "Performance indicator*" OR "performance measure*" OR "performance assessment*" OR "quality indicator*" OR "quality measure*" OR "quality system*" OR "quality evaluation" OR "quality assessment" )

AND

( healthcare OR "Health care" )

AND

( impact* OR effect* OR affect OR improve* )

AND

( nation* OR subnational OR sub-national OR "sub-national" OR govern* OR region* OR provinc* OR state* OR countr*)

AND

Articles

AND

English

**Search results:**

- Scopus: 3,423
- Isi web of science: 1,993
- Pubmed: 519
