# Supplementary material for: The contribution of benchmarking to quality improvement in healthcare. A systematic literature review
Source: BMC Health Serv Res. 2022 Feb 2;22:139. doi: 10.1186/s12913-022-07467-8 (PMC8812166; doi:10.1186/s12913-022-07467-8)
Supplement: Supplementary file 2 — Additional file 2. [file 12913_2022_7467_MOESM2_ESM.docx]

Additional file 2

Title: list of the variable included in the tool used to extract data

- Authors
- Article title
- Year of publication
- Title of the journal
- Abstract
- Clinical area
- Is quality improvement reported or not?
- Quality improvement activity (if reported)
- Scale of the benchmarking initiative
- Country/ies in which the benchmarking is taking place
- Number of units analysed in the article
- Type of participant adhesion (voluntary/mandatory)
- Type of benchmarking (Absolute/Relative)
- Benchmarking dimensions
- Use of incentives (yes/no)
- Reporting frequency
- Benchmarking developers
- Number of performance indicators analysed
- Communication of benchmarking results (public or not)
- Who’s in charge of the communication of benchmarking results?
- Type of study (empirical/conceptual)
- Methodology used (qualitative/quantitative)
- Research question
- Article’s main findings
