# Supplementary material for: The contribution of benchmarking to quality improvement in healthcare. A systematic literature review
Source: BMC Health Serv Res. 2022 Feb 2;22:139. doi: 10.1186/s12913-022-07467-8 (PMC8812166; doi:10.1186/s12913-022-07467-8)
Supplement: Supplementary file 4 — Additional file 4. [file 12913_2022_7467_MOESM4_ESM.docx]

Additional file 4

Title: Quality Assessment Tool (QATSDD)^[[1]](#footnote-1)^ scores for all reviewed articles

Item 1: Explicit theoretical framework

Item 2: Statement of aims/objectives in main report

Item 3: Clear description of research setting

Item 4: Evidence of sample size considered in terms of analysis

Item 5: Representative sample of target group of a reasonable size

Item 6: Description of procedure for data collection

Item 7: Rationale for choice of data collection tool(s)

Item 8: Detailed recruitment data

Item 9: Statistical assessment of reliability and validity of measurement tool(s) (Quantitative studies only)

Item 10: Fit between research question and method of data collection (Quantitative studies only)

Item 11: Fit between research question and format and content of data collection tool e.g. interview schedule (Qualitative studies only)

Item 12: Fit between research question and method of analysis (Quantitative studies only)

Item 13: Good justification for analytic method selected

Item 14: Assessment of reliability of analytic process (Qualitative studies only)

Item 15: Evidence of user involvement in design

Item 16: Strengths and limitations critically discussed

1. Sirriyeh, R., Lawton, R., Gardner, P., & Armitage, G. (2012). Reviewing studies with diverse designs: the development and evaluation of a new tool. *Journal of evaluation in clinical practice*, *18*(4), 746-752. [↑](#footnote-ref-1)
